# Reconstructive kidney surgery for organ-preserving therapy of renal tumors

**DOI:** 10.3205/iprs000069

**Published:** 2015-11-16

**Authors:** Amir Hamza, Manuel Günther, Wolf Behrendt, Stefan Tietze, Joachim Beige

**Affiliations:** 1 Department of Urology, Hospital St. Georg, Leipzig, Germany; 2 Department of Nephrology and KfH Renal Unit, Hospital St. Georg, Leipzig, Germany

**Keywords:** reconstructive kidney surgery, nephron sparing surgery, renal tumor, organ-preserving therapy

## Abstract

**Background: **The objective of this study was to evaluate differences in various clinical endpoints in patients with reconstructive surgery by renal partial nephrectomy for tumors up to 4 cm compared to tumors larger than 4 cm.

**Material and methods: **A total of 170 partial renal resection patients that presented malignant tumors were included in the retrospective study. Data was analyzed retrospectively based on internal clinic files, as well as a questionnaire to enhance the follow-up clinical outcomes data obtained. The most important outcomes determined included post-operative renal function, intra- and post-operative complications, local recurrence rate and total survival time.

**Results: **The local recurrence rate was 6.1% for tumors up to 4 cm in size, compared to 14.9% for tumors that were larger than 4 cm. Compared to results for partial resection of T1a tumors, results for partial resection of tumors larger than 4 cm are worse in terms of post-operative renal function (p=0.007), as well as in terms of a total complications rate (p=0.048). It is important to note that there was not only a higher risk of post-operative bleeding that required transfusions (p=0.012), but also a higher risk of a hypertensive episode during the post-operative period reviewed (p=0.022). In addition, the total survival time for patients presenting tumors of up to 4 cm in size was significantly better (p=0.003).

**Conclusion: **The results of our retrospective study of 170 patients that underwent partial renal resection after the diagnosis of malignant tumors, is that partial renal resection presents an oncologicaly safe surgical solution with low local recurrence rates. Additionally, partial resection in case of tumors that are larger than 4 cm showed worse post-operative renal function, a higher complications rate and a worse survival rate.

## Zusammenfassung

**Hintergrund: **Ziel dieser Studie war es, Unterschiede in verschiedenen klinischen Endpunkten bei Patienten mit rekonstruktiver Nierenchirurgie mittels Nierenteilresektion bei malignen Tumoren bis zu 4 cm beziehungsweise größer als 4 cm zu evaluieren.

**Material und Methoden: **Insgesamt wurden 170 Patienten mit einer Nierenteilresektion bei malignen Tumoren in die Studie eingeschlossen. Es wurde retrospektiv anhand der klinikinternen Unterlagen sowie eines Fragebogens zur Erhebung der Follow-up-Daten die klinischen Endpunkte erfasst. Wichtige klinische Endpunkte waren die postoperative Nierenfunktion, intra- und postoperative Komplikationen, die Lokalrezidivrate sowie das Gesamtüberleben.

**Ergebnisse: **Die Lokalrezidivrate betrug 6,1% bei Tumoren bis 4 cm bzw. 14,9% bei Tumoren größer als 4 cm. Im Vergleich zu T1a-Tumoren ist bei mehr als 4 cm großen Nierentumoren nach einer Nierenteilresektion mit einer schlechteren postoperativen Nierenfunktion (p=0,007) sowie mit einer höheren Gesamtkomplikationsrate zu rechnen (p=0,048). Insbesondere zeigte sich hier neben einem höheren Risiko einer transfusionspflichtigen Nachblutung (p=0,012) auch ein höheres Risiko einer hypertensiver Entgleisung im postoperativen Verlauf (p=0,022). Zudem war das Gesamtüberleben bei Tumoren bis 4 cm signifikant besser (p=0,003).

**Schlussfolgerung: **Den Ergebnissen unserer retrospektiven Studie nach, die 170 Pateinen mit Nierenteilresektion bei malignen Tumoren einschloss, ist die Nierenteilresektion ein onkologisch sicheres Operationsverfahren mit geringen Lokalrezidivraten. Allerdings ist bei Tumoren >4 cm mit einer schlechteren postoperativen Nierenfunktion, mit einer höheren Komplikationsrate sowie mit einem schlechterem Gesamtüberleben zu rechnen.

## Introduction

According to relevant guidelines, surgical treatment is still considered the only initial curative treatment for patients presenting localized renal cell carcinoma.

The first planned partial renal resection was performed by Vinzenz Czerny in Heidelberg toward the end of the 1900s [1]. Since the first procedure, the technique developed progressively and indications increased correspondingly. Sparing organs in cases of T1 tumors has been an established preference for some time in surgical routines and is a prerequisite anchored in international guidelines [2]. The EAU Guidelines recommend parenchymal conservation for partial renal resection, when technically possible, in case of tumors that are smaller than 4 cm, as well as for tumors that are between 4 and 7 cm in size [2].

As a result of increasing routine imaging procedures such as ultrasonography and tomography, there is an increased level of discovering the incidence of T1 tumors [3]. It therefore became essential to improve surgical renal preservation measures. Organ conservation benefits include maintaining renal functions, preventing renal insufficiencies and reducing fatal cardiovascular risks [4]. Compared to tumor nephrectomy, it remains a sophisticated technical procedure with high complication rates. In addition, only T1a tumors allow clear final oncological certainty. Even after nephrectomy, up to 20% show late recurrence [5].

Aside from the elective indications related to T1a tumors and healthy contra-lateral kidney, there are relative indications for a higher risk of deteriorating renal function in cases of any or more of the following: reflux, diabetes mellitus and arterial hypertonia. Absolute indications for renal conservation are single kidneys, bilateral tumors and existing renal insufficiency [6]. The Nephrometry Scores (such as R.E.N.A.L.) classify the complexity of renal tumors for consideration. Additional factors to be considered include typical characteristics such as diameter, exophytic characteristics, distance from inner cavity and localization (position from pole line, anterior or posterior position). Irrespective of these aspects, it is impossible to estimate post-operative renal function reliably.

Tumor removal is possible within segment/pole resection or simple enucleation. In such cases, it is adequate to maintain a slight safety distance [7]. Apart from the open access in retro- or transperitoneal surgery, the European guidelines include laparoscopic and robot assisted surgical techniques as alternative procedures. 

In cases of large and centrally situated tumors, exact resection may require ischemia, but in case of warm ischemia, the ischemia time should not exceed 30 minutes [8]. Wider use of haemostyptics could contribute to improved hemostasis and lead to reduced blood loss. This first contributed to renal conservation in cases of larger tumors. 

## Objective

The objective of the work is to research and ascertain the oncological outcomes of partial renal resection in patients presenting renal cell carcinoma, based on specific parameters and depending on tumor stage. 

## Material and methods

This retrospective analysis relates to 170 patients that underwent partial renal resection at our clinic. Each patient presented histologically confirmed renal cell carcinoma and all patients included in this study were treated from January 2010 to December 2013. We collected and analyzed the patient data based on patient files, surgical reports, anesthetics protocol and histological findings. 

Indications for partial renal resection were determined according to the general health of the patients, pre-operative renal functions, position of the tumor and tumor stage. Two distinct patient groups were identified. One group presented a tumor size (T) of up to 4 cm (T1a, n=107) and the other group presented renal tumors that were larger than 4 cm (T1b–T2, n=63).

GFR (glomerular filtration rate) was determined pre- and post-operatively based on creatinine levels as defined by Cockcroft-Gault. Complications were recorded according to their nature and frequency and compared in the respective groups. The study also recorded surgery time and duration of stay in the intermediate care unit (IMC). 

The total survival rate, recurrence, newly emerged cardiovascular conditions and renal function were documented post-operatively for a period of up to 54 months. A questionnaire helped to obtain data about the treated patients. The average follow-up period was 29 months after the intervention. 

SPSS (Version 21) was used for statistical evaluation, with the application of T tests, Mann-Whitney u and Chi-Square tests. The statistical significance determined was p=0.05.

At our clinic we applied both open transperitoneal and laparoscopic techniques for partial renal resection. Depending on position and size of the tumors, a double J endo-ureter catheter was placed peri-operatively to assist with excretion from the inner cavity. 

**Figure 1:**
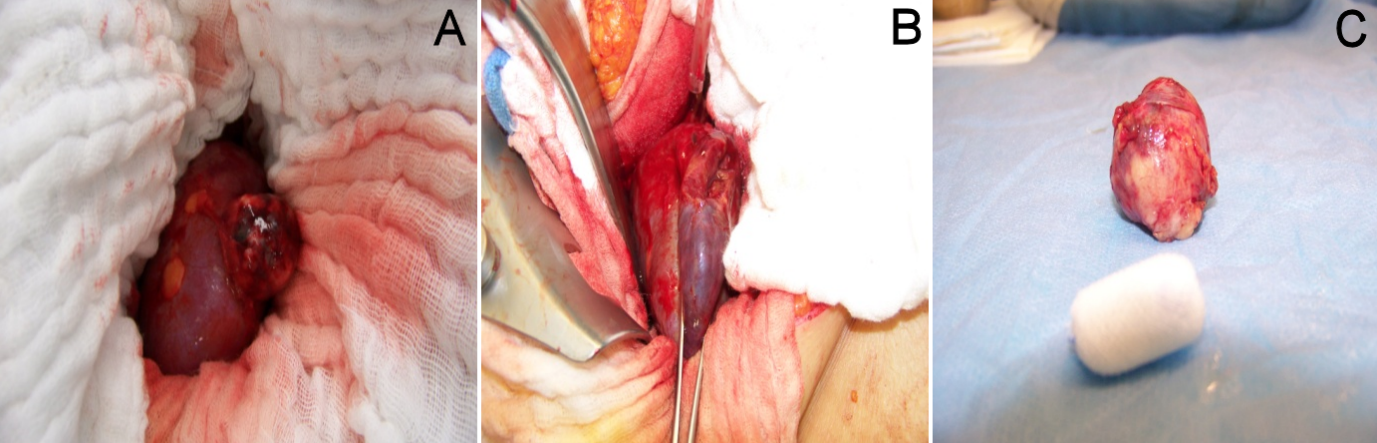
Open partial renal resection with partial warm ischemia

**Figure 2:**
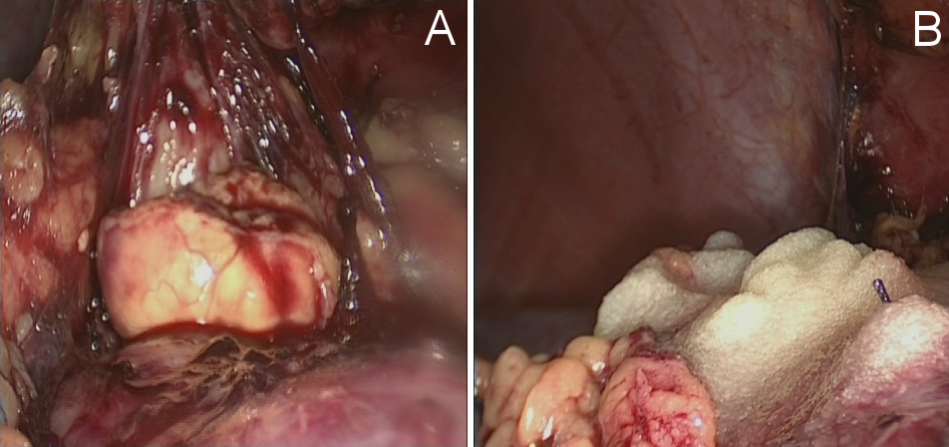
Laparoscopic partial renal resection technique

The next step was the complete preparation of the renal surface (Figure 1A (Fig. 1) and Figure 2A (Fig. 2)) with exposure of the renal vessels. A special soft intestinal clamp was used to induce partial ischemia to the areas affected by the tumor (Figure 1B (Fig. 1)). Larger central tumors were resected during complete warm ischemia. After excision (Figure 1C (Fig. 1)), a quick surgical diagnosis of the resection area was performed. At the opening of pyelocaliceal system the plastic reconstruction of the renal pelvis was performed with vicryl 4/0 suture material. Repairs to the resection area were performed using parenchymal sutures. In case of slight and superficial bleeding it was not necessary to apply hemostyptics to the parenchymal sutures (Figure 2B (Fig. 2)). 

The post-operative management involved close monitoring for at least one day in the IMC and prescribed bed rest for three days. The urinary catheter was removed after seven days in cases without obvious deviation from clinical expectations.

## Results

The average patient age was 66 years. Of the patients, 94 (55.3%) were male and 76 (44.7%) were female. The percentage of male patients presenting a ≤4 cm tumor was 56.1% and those with a >4 cm tumor 54.0%. Among female patients those with ≤4 cm tumors represented 43.9% while those with >4 cm tumors represented 46%.

### Renal function

A comparison of pre- and post-operative GFR showed a significantly larger reduction in the T >4 cm group. Both the difference between pre- and post-operative serum creatinine and the glomerular filtration rate showed significant variances between the T ≤4 cm group and the T >4 cm group. 

The same result was shown in the comparison of pre-operative serum creatinine and the serum creatinine checked during the follow-ups (p=0.021). 

Follow-up investigations showed a response rate of 79.4% (n=135). A few individual questionnaires were not completed in full, explaining the respective variable follow-up figures (see Table 1 (Tab. 1)).

### OP and hospitalization duration 

No significant differences were observed between the two groups in terms of OP duration or post-operative duration of monitoring in the IMC. 

### Complications rate

A higher complications rate was recorded for the T >4 cm group. The rates for post-operative bleeding that required transfusions and newly emerging hypertensive episodes were significantly higher. 

The other investigation parameters such as the revision rate, the occurrence of post-operative renal failure, hematoma and urinoma formation, problematic wound healing and the formation of abscesses showed no significant differences. 

### Nephrectomy after partial renal resection

During the period investigated, no nephrectomies due to uncontrollable complications were required for patients during the initial stationary in-patient period. Over the total period reviewed, the number of patients requiring an ipsi-lateral nephrectomy after a partial renal resection showed a significantly higher nephrectomy rate in the T >4 cm group. In the latter group 12.5% of patients required a nephrectomy compared to only 2.9% in the T ≤4 cm group (see Table 2 (Tab. 2)). The number includes nephrectomies due to local recurrence, as well as nephrectomies that were required after late-onset complications.

### Survival rate

The survival rate in the follow-up investigation showed a significantly longer survival rate in the T <4 cm tumor group. At 29 months after the partial renal resection intervention for patients with tumors of up to 4 cm, 97.6% of patients survived. In contrast, the total survival rate for patients in the T >4 cm group was 84.0% (see Table 3 (Tab. 3)).

### Local recurrence

Another objective of the investigation was to determine local recurrences within the specific time period. According to the data 5 (6.1%) of the patients in the group with tumors up to 4 cm experienced local recurrence, compared to 7 (14.9%) in the T >4 cm group. The difference was not significant (see Table 3 (Tab. 3)). 

### Newly developed arterial hypertonia and coronary heart disease

During the period reviewed, three patients developed coronary heart disease or arterial hypertonia. There was no significant difference between the groups (see Table 3 (Tab. 3)). 

## Discussion

### Renal function and newly developed arterial hypertonia/coronary heart disease

Generally both groups showed deteriorating post-operative renal function. It could be ascribed to the loss of functional renal tissue. It was also noted that compared to smaller tumors, the larger tumors resulted in increased deteriorating renal function, measured in terms of serum creatinine. A later improvement in renal value, as described by Gratzke et al. [9], was not observed. Our research showed an acceptable reduction in renal function without the development of renal insufficiency that requires dialysis. The study by Go et al. [10] showed a clear increased risk of cardiovascular disease that correlates with deteriorating renal function. Our study showed newly developed cardiovascular disease in three patients, but no long-term increase in incidence was recorded. It may be ascribed to the slight deterioration of renal function. 

### Complications

The complication rates after partial renal resection described in the literature showed a high variability of 10% [11] to 30.7% [12]. These also depended on the set indications. Imperative indications clearly involve higher complication rates than elective indications. These rates remain between 15% and 20% [13], [14]. In our investigation the total complication rates were a little higher than those recorded in the literature (see Table 2 (Tab. 2)). A closer look at the differences shows that all complications, for both groups, were controllable without requiring a direct nephrectomy. The complications rate in our investigation was, like the rates described in other investigations [15], significantly higher in the T >4 cm group. 

According to the literature, intra-operative blood loss varied between 335 ml [16] and 494 ml [9]. Our investigation did not determine intra-operative blood loss due to a number of factors that could have affected the results, such as blood lost into abdominal swabs, compresses or other swabs and blood retained in tumor preparations. However, the individual transfusion requirements were evaluated based on the hemoglobin values recorded during the period. These showed a significantly higher transfusion rate in the T >4 cm group. At 14.3% the rate is low compared to other investigations [17], that showed transfusion rates of up to 30%.

### Oncological outcome

Partial renal resection is considered an oncologicaly safe treatment for T1a tumors. The literature shows comparative equal values at 98% for tumor specific five-year survival rates, when compared to those for radical nephrectomy [18]. Indicated local recurrence rates in a five-year follow-up relating to T1a tumors are between 2.7% and 3% [18], [19]. Organ conservation resection of tumors larger than 4 cm showed a five-year tumor specific survival rate of between 85% and 98% [20], [21]. Our data confirms the survival rates, although it is important to note that our follow-up investigation related to 29 months. The local recurrence rate recorded was 6.1% (T ≤4 cm) and 14.9% (T >4 cm), clearly higher. These resulted in a total of 10 nephrectomies within the follow-up period. 

## Conclusion

Organ preserving partial renal resection in case of T1a tumors was the subject of our investigation into oncologicaly safe surgical procedures and results showed a small recurrence rate and high survival rate after 29 months.

Our investigation compared results for partial renal resection of renal tumors of up to 4 cm in size and tumors that are larger than 4 cm. Results for the latter were worse in terms of post-operative renal function and showed a higher complications rate during the course of the post-operative period reviewed, as well as a reduced total survival rate. As such, partial renal resection of tumors larger than 4 cm should depend on age, patient morbidity and complexity of the tumor. 

## Notes

### Competing interests

The authors declare that they have no competing interests.

## Figures and Tables

**Table 1:**
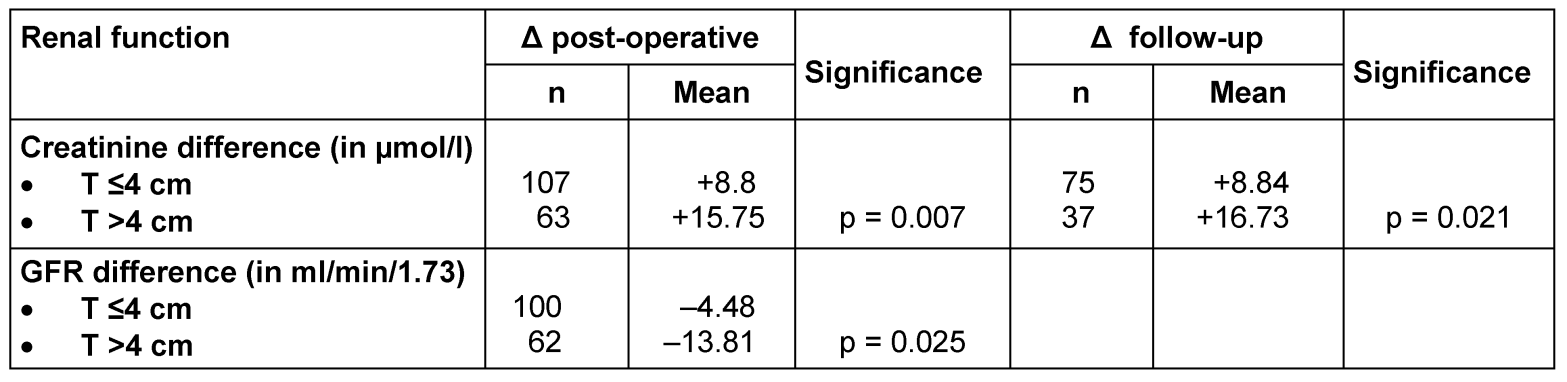
Renal function development after partial renal resection depending on tumor size

**Table 2:**
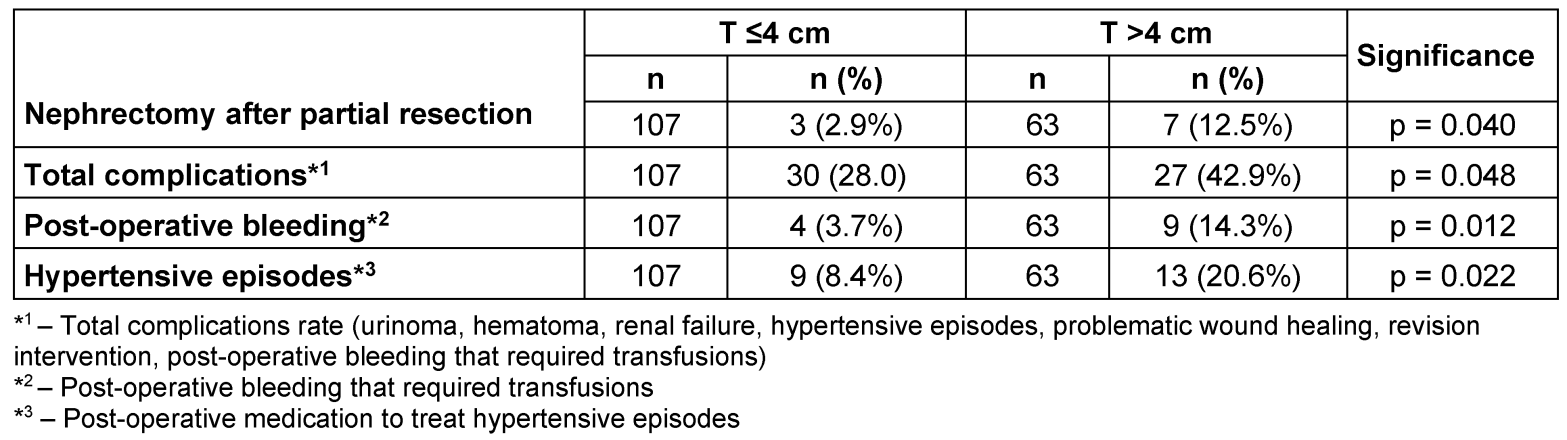
Post-operative complications

**Table 3:**
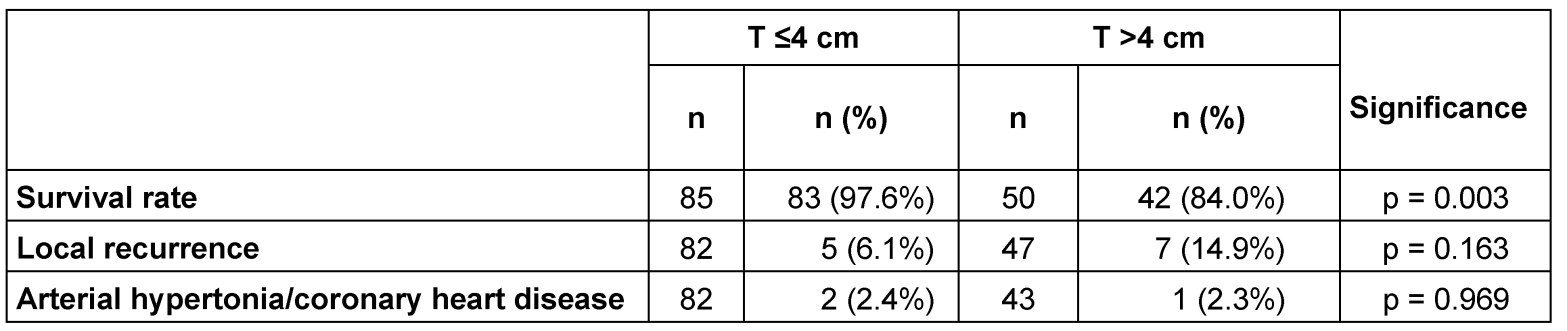
Results of the follow-up investigation relating to the 29 months after the intervention
